# Role of Deficient Mismatch Repair in the Personalized Management of Colorectal Cancer

**DOI:** 10.3390/ijerph13090892

**Published:** 2016-09-08

**Authors:** Cong-Min Zhang, Jin-Feng Lv, Liang Gong, Lin-Yu Yu, Xiao-Ping Chen, Hong-Hao Zhou, Lan Fan

**Affiliations:** 1 Department of Clinical Pharmacology, Xiangya Hospital, Central South University, Changsha 410008, China; ZhangCM126126@126.com (C.-M.Z.); gongliang3991@163.com (L.G.); yulinhui19910530@163.com (L.-Y.Y.); chenxp74@hotmail.com (X.-P.C.); hhzhou2003@163.com (H.-H.Z.); 2 Institute of Clinical Pharmacology, Central South University, Hunan Key Laboratory of Pharmacogenetics, Changsha 410078, China; 3 Department of Pharmacy, Xiangya Hospital, Central South University, Changsha 410008, China; lvjinfeng1990@163.com; 4 Institute of Hospital Pharmacy, Central South University, Changsha 410008, China

**Keywords:** colorectal cancer, personalized treatment, mismatch repair system, microsatellite instability, adjuvant chemotherapy, targeted therapy, immune checkpoint inhibitor therapy

## Abstract

Colorectal cancer (CRC) represents the third most common type of cancer in developed countries and one of the leading causes of cancer deaths worldwide. Personalized management of CRC has gained increasing attention since there are large inter-individual variations in the prognosis and response to drugs used to treat CRC owing to molecular heterogeneity. Approximately 15% of CRCs are caused by deficient mismatch repair (dMMR) characterized by microsatellite instability (MSI) phenotype. The present review is aimed at highlighting the role of MMR status in informing prognosis and personalized treatment of CRC including adjuvant chemotherapy, targeted therapy, and immune checkpoint inhibitor therapy to guide the individualized therapy of CRC.

## 1. Introduction

Colorectal cancer (CRC) represents the third most common type of cancer in developed countries and one of the leading causes of cancer deaths worldwide [1]. For primary CRC, the tumor-node-metastasis (TNM) stage is considered the gold standard for informing prognosis and treatment after resection [2]. Nevertheless, it is fair to admit that there is considerable stage-independent variability in the clinical outcome of CRC, which might result from molecular heterogeneity of the tumors [3]. Hence, the identification of molecular markers for prognosis and treatment of CRC is urgently needed to achieve personalized management of CRC. 

It has been shown that there are two recognized pathways contributing to CRC development [4]. The majority of CRCs develop via a chromosomal instability pathway (CIN), and approximately 15% are caused by deficient mismatch repair (dMMR) [4]. Molecular heterogeneity is commonly considered pivotal to guide the management of CRCs [5]. Coincident data demonstrated that compared with those with proficient MMR (pMMR) tumors, dMMR CRC patients had better stage-adjusted clinical outcome [6,7] and might benefit differently from a variety of therapies including adjuvant chemotherapy (fluoropyrimidine, platinum compounds, topoisomerase inhibitors, and alkylating agents), targeted therapy (anti-epithelial growth factor receptor (EGFR) or anti-vascular endothelial growth factor (VEGF) antibodies) and immune checkpoint inhibitor therapy (anti-programmed cell death 1 (PD-1) antibodies) [2,3,8,9]. Here we discuss the prognostic and predictive value of MMR status in the clinical personalized management of CRC.

## 2. dMMR in CRC

### 2.1. MMR System, dMMR, and Microsatellite Instability (MSI) Phenotype

DNA synthesis is an error-prone process which generates incorrect base-pairing (base-base mismatches) or unmatched DNA loops (insertion-deletion loops). As one of the DNA repair mechanisms, the MMR system can repair these errors to maintain genomic stability. The MMR system is composed of a series of MMR proteins including MutL homolog 1 (MLH1), MutL homolog 3 (MLH3), MutS homolog 2 (MSH2), MutS homolog 3 (MSH3), MutS homolog 6 (MSH6), postmeiotic segregation increased 1 (PMS1), and postmeiotic segregation increased 2 (PMS2) [10]. These MMR proteins function by forming heterodimer complexes MutS and MutL. Consisting of two major forms MutSα (a MSH2/MSH6 heterodimer) and MutSβ (a MSH2/MSH3 heterodimer), MutS performs the initial recognition of mismatches [11]. MutSα recognizes and binds to base-base mismatches and short (1–2) insertion deletion loops (IDLs), whereas MutSβ detects larger (≥2) IDLs [9]. Then, MutL together with other repair proteins including PCNA (proliferating-cell-nuclear-antigen) and exonucleases completes the DNA repair process [9,11]. MutL homologs include MutLα (a MLH1/PMS2 heterodimer), MutLβ (a MLH1/PMS1 heterodimer), and MutLγ (a MLH1/MLH3 heterodimer) [11]. MutLα plays a more major role in DNA mismatch repair compared with the other two [12]. MMR genes generally act as tumor suppressor genes. dMMR commonly results from a consequence of germline mutations in MMR genes, somatic MMR gene alterations, or epigenetic silencing of MMR gene expression [13].

Microsatellites are short (1–6 base pairs), tandem repeated sequences that are scattered throughout the genome and very susceptive to replication errors induced by the slippage of DNA polymerases [14]. Generally, these errors can be corrected by the MMR system to keep the stability of microsatellites. When the MMR system is deficient owing to genetic or epigenetic events, tumors exhibit MSI phenotype. Thus, it is a well-established concept that MSI serves as a phenotypic indicator of dMMR. 

### 2.2. dMMR CRC

dMMR CRCs account for about 15% of all primary CRCs [13] including sporadic CRCs (12%) and Lynch syndrome (LS) (3%) [15]. CRC patients with dMMR tumors have unique clinicopathological features such as proximal colon preponderance, poor differentiation, early stage and abundant tumor-infiltrating lymphocytes compared with those displaying pMMR tumors (Box 1).

Box 1 **Clinicopathological features of dMMR tumors [16,17,18,19,20].**

1. Proximal Colon Predominance (70% Proximal to the Splenic Flexure)
2. Poor Differentiation
3. Tumor Heterogeneity
4. Large and Lymph-Node-negative
5. Excess of Mucinous (15%), Signet Cell and Medullary Subtypes
6. Prominent Anti-tumor Host Response (Increased Tumor-Infiltrating Lymphocytes as Well as “Crohn-like” Reaction)
7. Accelerated Carcinogenesis from Tiny Adenoma to Carcinoma within 2–3 Years in Lynch Syndrome Cases

Abbreviation: dMMR, deficient mismatch repair.

#### 2.2.1. dMMR in LS

LS is an inherited autosomal-dominant disorder, also known as hereditary nonpolyposis colorectal cancer (HNPCC). The development of LS requires ”two hit“ inactivation of both alleles of the MMR gene. LS is caused by a germline inactivating mutation in one of the MMR genes, commonly *MLH1* or *MSH2*, infrequently *MSH6* or *PMS2* (the first hit). Subsequently, the remaining allele would lose function through somatic mutation, loss of heterozygosity (LOH) or promoter methylation (the second hit) leading to LS [13]. In addition, germline mutation in the epithelial cell adhesion molecule (*EPCAM*), a gene located upstream of *MSH2* can cause epigenetic inactivation of *MSH2* leading to LS [21]. LS patients are diagnosed at an earlier age, and are at high risk of various cancers such as stomach cancer, ovary cancer, urinary tract cancer, small intestine cancer, and prostate cancer. The revised Bethesda Guidelines (RBG) was developed to identify individuals at risk of LS by testing for dMMR/MSI of tumors (Box 2).

Box 2 **Revised Bethesda Guidelines for MSI testing [22,23,24,25].**

1. CRC Diagnosed in a Patient Younger than 50 Years
2. Presence of Synchronous or Metachronous CRC or Other Lynch Syndrome-Associated Tumor *, Regardless of Age
3. CRC with MSI-H Pathological Features **^#^** Diagnosed in a Patient Younger than 60 Years
4. Patient with CRC and CRC or Lynch Syndrome-associated Tumor * Diagnosed in at Least One First-Degree Relative Less than 50 Years of Age
5. Patient with CRC and CRC or Lynch Syndrome-Associated Tumor * Diagnosed in Two or More First-Degree or Second-Degree Relatives, Regardless of Age

* Lynch syndrome-associated tumors include cancers of colorectum, endometrium, stomach, ovary, pancreas, biliary tract, small bowel, ureter, renal pelvis, and brain tumors, as well as sebaceous gland adenomas and keratoacanthomas. **^#^** MSI-H pathological features include tumor infiltrating lymphocytes, Crohn-like lymphocytic reaction, and mucinous or signet-ring cell differentiation, or medullary growth pattern. Abbreviations: CRC, colorectal cancer; MSI, microsatellite instability.

#### 2.2.2. dMMR in Sporadic CRC

In sporadic CRCs, dMMR occurs more frequently in stage II (~20%) and stage III (~12%) tumors, and very rare in metastatic cases (~4%), which indicates that dMMR CRC is less metastatic and MMR status detection in earlier stage is of great importance [26,27]. The vast majority of sporadic CRCs are caused by suppression of MLH1 expression (~95%) due to hypermethylation of the *MLH1* promoter known as the CpG island methylator phenotype (CIMP) [28,29], and inactivation of *MSH2* and *MSH6* account for the small percentage (~5% and ~1%) respectively. About half of sporadic dMMR cases carry *BRAF* V600E mutations which could distinguish sporadic tumors from LS cases [30,31]. Although sharing many similar features, dMMR sporadic CRCs have different clinical characteristics compared with LS, including older age and female predominance [19]. 

## 3. Identification of dMMR/MSI in CRC Tumors

There are two broadly accepted methods for dMMR/MSI detection including MSI testing and MMR protein expression analysis by immunohistochemistry (IHC). It has been shown that MSI testing and IHC are complimentary and the result of MMR proteins expression by IHC is concordant with DNA based MSI testing with a favorable sensitivity and a dramatic specificity [32,33]. IHC is commonly used as an alternative test when a molecular laboratory is not available and is able to pinpoint the affected gene by detecting its protein expression assisting in identifying patients with LS [33]. 

### 3.1. MSI Testing

MSI testing is performed by comparing allelic profiles of microsatellite markers in tumor tissue DNA with matching normal DNA from each patient through a PCR-based assay [33]. A panel of five microsatellite sequences, known as the Bethesda panel has been validated and recommended as a reference panel including 2 mononucleotide repeats (BAT26 and BAT25) and 3 dinucleotide repeats (D2S123, D5S346 and D17S250) [34]. According to these microsatellite markers, CRCs can be classified into three groups: MSI-H with two or more of the five microsatellite markers showing instability, MSI-L (low-frequency MSI) with only one of five markers showing instability and MSS (microsatellite stable) with none of the five markers showing instability. Notably, the 2002 National Cancer Institute Workshop made a revision recommending a secondary panel of microsatellite markers with mononucleotide repeats such as BAT-40 and/or MYCL to exclude MSI-L tumors in which only dinucleotide repeats were mutated [25]. 

### 3.2. MMR Protein Expression Detection by IHC 

Using IHC, tumors exhibiting loss of a MMR protein are considered as dMMR/MSI and those with intact MMR proteins are classified as pMMR/MSS or MSI-L. Absent expression of a MMR protein (MLH1, MSH2, MSH6 or PMS2) can guide a follow-up germline test to find out the affected gene to screen for LS. LS cases can be diagnosed by isolated loss of MSH2 or MSH6 protein. MLH1 and PMS2 proteins are commonly lost concurrently, and so are MSH2 and MSH6 proteins [15]. Isolated loss of MLH1 protein has been described in sporadic CRCs [35]. In addition, CRCs with loss of MLH1 protein expression are always advised to detect *BRAF* mutations to confirm sporadic cases [36].

## 4. Predictive Value of MMR Status in CRC Treatment

### 4.1. Chemotherapeutic Agents

#### 4.1.1. Fluoropyrimidine

A fluoropyrimidine (5-FU or capecitabine)-based adjuvant chemotherapy is considered as standard care for selected stage II and stage III CRC after surgery [37,38]. Clinicians commonly choose intravenous 5-FU or oral prodrug capecitabine or combine them with other chemotherapeutic agents such as irenotecan, leucovorin, and oxaliplatin to increase the response rate (RR) [39]. 5-FU functions by conversion to a series of active metabolites including fluorodeoxyuridine monophosphate (FdUMP), fluorodeoxyuridine triphosphate (FdUTP), and fluorodine triphosphate (FUTP), which kill tumor cells in different mechanisms. FdUMP inhibits thymidylate synthetase (TS), a pivotal element in generating nucleotides required for DNA replication, whilst FdUTP and FUTP incorporate into DNA and RNA respectively [40]. FUTP incorporation has been considered cytotoxic because it disrupts RNA processing while FdUTP lesion in DNA would be lethal by contributing to DNA strand breaks or apoptosis [40]. 

Although not all preclinical studies agreed [41,42], most supported that dMMR was associated with poor benefit from 5-FU treatment in CRC [43,44,45,46]. Carethers et al. [44] reported that HCT116 (a MLH1 deficient CRC cell line) was approximately 18-fold more resistant to 5-FU compared with pMMR cells. When introduced by chromosome 3 transfer containing the *MLH1* gene, HCT116 was sensitive to 5-FU treatment [44]. Meyers et al. [45] obtained similar results in another study both in HCT116 and HEC59 (a MSH2 deficient human endometrial adenocarcinoma cell line). DNA mismatches produced by insertion of 5FdUTP into DNA could not be recognized in dMMR cells, which led to cell survival and drug resistance [47,48,49,50], and when MMR deficiency was corrected, resistance to 5-FU was reversed [49]. A MSH2 deficient xenograft experiment conducted by Pocard et al. also showed that dMMR CRC was resistant to 5-FU [51].

There are many clinical studies about the relationship between MMR and the response of CRC to 5-FU (Table 1). Abundant clinical data suggested that 5-FU-based adjuvant chemotherapy was ineffective in CRC patients with dMMR tumors. A retrospective analysis carried out by Ribic et al. [52] found that pMMR status was significantly correlated with increased overall survival (OS) (hazard ratio (HR), 0.72; 95% confidence interval (CI), 0.53–0.99) among stage II/III patients who received FU-based adjuvant chemotherapy, while dMMR CRC patients experienced no benefit. Another prospective analysis of data from randomized, clinical trials by Sargent et al. [53] demonstrated that no benefit from 5-FU treatment was observed for patients with either stage II (HR = 2.30; 95% CI = 0.85–6.24; *p* = 0.09) or stage III (HR = 1.01; 95% CI = 0.41–2.51; *p* = 0.98) dMMR CRCs. However, some studies considered that dMMR CRCs derived a similar or even a greater benefit from 5-FU-based adjuvant treatment compared with pMMR CRCs [54,55,56,57]. Conflicting results were likely due to the bimodal age distribution of CRC patients, limited sample size, inclusion of multiple tumor stages and different 5-FU-based adjuvant regimens etc. [58,59]. 

#### 4.1.2. Platinum Compounds

Platinum compounds such as carboplatin, cisplatin, and oxaliplatin play an important role in chemotherapeutic treatment of many malignant tumors [63]. These platinum-containing drugs kill cancer cells by forming DNA adducts contributing to intrastrand or interstrand cross-links, which change the DNA molecule leading to cell cycle arrest and apoptosis [63]. 

Unfortunately, intrinsic or acquired drug resistance has limited the usage of platinum compounds in most cancers including CRC. Aebi et al. [42] showed that two dMMR tumor cells, HCT116 and HEC59 were both resistant to cisplatin and carboplatin compared with pMMR subline HCT116 + ch3 cell (complemented with chromosome 3 bearing the wild-type gene for MLH1) and pMMR subline HEC59 + ch2 cell (complemented with chromosome 2 bearing the wild-type gene for MSH2). The mechanism involved was that platinum complexes could interfere with the functional MMR system to stop a complete repair of DNA damage leading to cell apoptosis [64]. When MMR was deficient, cells could proliferate with DNA damage induced by platinating agents and drug resistance would occur. Thus, dMMR is a key determinant in resistance of CRC cells to cisplatin and carboplatin management. 

However, it was shown that MMR deficiency seemed not to confer drug resistance to a new platinum compound oxaliplatin. A pooled analysis of the NSABP-C07 and NSABP-C08 trials demonstrated that the benefit of adding oxaliplatin to 5-FU was not associated with MMR status [65]. Meanwhile, several retrospective studies reported that adding oxaliplatin to 5-FU could restore the efficacy of adjuvant chemotherapy in stage III dMMR CRCs [38,66,67]. A retrospective study carried out by Zaanan et al. showed that addition of oxaliplatin to 5-fluorouracil and leucovorin (FL) significantly improved disease-free survival (DFS) of stage III CRC patients with dMMR tumors (HR 0.17; 95% CI = 0.04–0.68; *p* = 0.01) [66]. Flejou et al. [68] were also in favor of FOLFOX4 (a fluorouracil, leucovorin, and oxaliplatin regimen) vs. LV5FU2 (a fluorouracil and leucovorin regimen) in dMMR CRC patients. The reason why dMMR CRC cells were sensitive to oxaliplatin was that the special 1, 2-diaminocyclohexane (DACH) ligand of oxaliplatin prevented MMR complex from binding to its DNA adducts, which led to failure of repair and subsequent apoptosis of tumor cells [69]. Thus oxaliplatin had efficacy in CRC cells resistant to cisplatin and carboplatin [42,69,70].

#### 4.1.3. Topoisomerase I Inhibitors

Camptothecin (CPT) and its derivative irinotecan (CPT-11) are topoisomerase I inhibitors that induce transient DNA single-strand breaks which are subsequently converted into permanent DNA double strand breaks (DSB) and ultimately cause cell apoptosis. CPT-11 has been commonly applied in the treatment of metastatic CRC (mCRC) as an effective complement for 5-FU.

When it comes to the relationship between MMR status and the response of CRC to CPT-11, there has been a long-term controversy. There appeared to be strong preclinical evidence indicating that dMMR CRC tumors were more sensitive to CPT-11 treatment than pMMR tumors [71,72]. Magrini et al. [73] suggested the percentage of apoptotic cells after treatment with CPT-11 was higher in dMMR CRC cells than that in pMMR cells, suggesting that an intact MMR system might prevent CPT-11-induced apoptosis. A recent research showed that dMMR CRC cells were more sensitive to CPT-11 and MMR status might be a predictive biomarker of response to CPT-11-based chemotherapy in mCRC [74]. The reasons below might explain the fact that dMMR CRC tumors were more sensitive to CPT-11 treatment: Firstly, when the MMR system was defected, the DSB induced by CPT-11 could not be repaired and the apoptosis of tumor cells occurred [74]. Secondly, dMMR CRC cells commonly generated secondary mutations in DSB repair genes such as MRE11A and hRAD50, which might have improved the efficacy of CPT-11 [75]. A few studies showed that dMMR CRC cells were resistant to camptothecin derivatives [76,77], which might have resulted from different detection methods such as the clonogenic assay and MTT test.

As shown in Table 2, the clinical results about the relationship between MMR status and CPT-11 were quite inconclusive. Some studies found that dMMR was associated with better clinical outcome to CPT-11 management in mCRC patients. A retrospective study of 72 mCRC patients conducted by Fallik et al. [78] found that dMMR tumors experienced improved RR to CPT-11 compared with pMMR tumors (57.1% vs. 10.8%). Charara et al. [79] demonstrated that dMMR was predictive of an improved response to neoadjuvant chemotherapy containing CPT-11 and radiation therapy in early stage rectal cancer. Similarly, Bertagnolli et al. [80] showed that CRC patients with dMMR tumors had improved 5-year DFS as compared with pMMR tumors when treated with irinotecan, FU, and leucovorin (IFL). However, there were clinical data indicating that MMR status was not associated with the response to CPT-11 in CRC treatment [6,81,82]. These varied conclusions might be due to the fact that most studies were small-scale, retrospective, or nonrandomized, with a significant bias as well as the different CPT-11-based regimens involved in the trials [6]. For instance, CPT-11 was often used in combination with 5-FU, to which dMMR cells were resistant. Hence, further studies are definitely needed to confirm the efficacy of CPT-11 in dMMR tumors to guide its individual administration in CRC subsets. 

#### 4.1.4. Alkylating Agents

Alkylating agents including *N*-Methyl-*N*-nitrosurea (MNU), *N*-methyl-*N*’-nitro-*N*-nitrosoguanidine (MNNG), procarbazine, and temozolomide (an activated form of procarbazine) induce various adducts on DNA, notably *O*^6^-methylguanine [83]. *O*^6^-methylguanine DNA methyltransferase (MGMT) possesses the ability to remove the carcinogenic *O*^6^-methylguanine (*O*^6^-MeG) DNA adducts and has been demonstrated to serve as a biomarker to define the likely benefit from alkylating agents [84]. Some in vitro experiments highlighted that the MMR system was a pivotal determinant in the response of CRC cells to alkylating agents. The dMMR CRC cell line HCT116 was found to be tolerant to MNNG and the resistance phenomenon could be reversed when the MMR defect was corrected [85]. A similar result was shown in another CRC cell LOVO, which contained a mutated *MSH2* gene from exon 3 to exon 8 [86]. The underlying mechanism was probably that a competent MMR system was necessary for G2 arrest after alkylating agent treatment, and dMMR tumor cells failed to undergo this arrest [87]. Also, MMR proteins could recognize lethal DNA adducts which were neglected by MGMT, and then initiated an apoptotic progress contributing to cell death [88]. In conclusion, sensitivity to alkylating agents likely requires taking both the MGMT expression and the functional MMR system into consideration. 

To date, little clinical trial data describing the role of dMMR in response to alkylating agents in CRC has been reported. A phase II study of temozolomide found that all 5 pMMR CRC patients were with partial responses (PR) and 83% dMMR CRC patients had progressive disease, suggesting that a proficient MMR system seemed to be required for the response to temozolomide, but the overall RR (6%) was too low to permit this conclusion [89]. 

### 4.2. Targeted Therapy 

#### 4.2.1. Anti-EGFR Targeted Therapy

EGFR is a receptor tyrosine kinase involved in the development and metastasis of CRC. Monoclonal antibodies (mAbs) targeting EGFR including cetuximab and panitumumab are used alone or in combination with chemotherapy in mCRC treatment. *KRAS* or *BRAF* mutations in mCRCs have been identified as well validated markers of a poor response to EGFR-targeted antibodies [90,91,92]. Previous works demonstrated that *BRAF* mutations were significantly associated with dMMR CRCs especially sporadic dMMR cases. Wang et al. [93] found that 34% dMMR CRCs had *BRAF* mutations and most occurred in those with promoter hypermethylation of *MLH1*, whereas only 12% pMMR CRC displayed *BRAF* mutations. Tran et al. [94] also showed that *BRAF* mutations were significantly more common in dMMR CRC tumors. Therefore, whether dMMR mCRC could benefit more from anti-EGFR targeted therapy deserves further exploration. 

#### 4.2.2. Anti-VEGF Targeted Therapy

VEGF, a potent regulator of physiologic and pathologic angiogenesis plays an essential role in tumor progression and metastasis. VEGF-A has been proven not only to suppress the maturation of dendritic cells (DCs) [95] and T cells [96] but also to alter the function of dendritic cells potently generating a tumor-associated immune suppression [97]. Moreover, VEGF-A could directly induce regulatory T cells (Tregs) proliferation in tumor-bearing mice through VEGF-A/VEGFR pathway [98], and specific blockade of this pathway could prevent the accumulation of Tregs in tumor-bearing mice and mCRC patients [98]. 

Bevacizumab in combination with chemotherapy exhibited attractive clinical activity in mCRC patients [99]. A retrospective analysis by Pogue-Geile et al. [100] revealed that stage II/III dMMR CRC patients significantly derived potential survival benefit from the addition of bevacizumab to standard FOLFOX (HR = 0.52; 95% CI = 0.29–0.94) in contrast to pMMR patients (HR = 1.03; 95% CI = 0.84–1.27). Hansen et al. [101] suggested that CRC patients with dMMR tumors had higher serum VEGF-A levels than those with pMMR tumors. Additionally, adding bevacizumab to adjuvant chemotherapy induced a decrease in Treg percentages but not conventional T cells in the peripheral blood of mCRCs [101]. Thus dMMR CRC patients might get significant clinical benefit from bevacizumab owing to their immunosuppressive microenvironment and high serum levels of VEGF-A. These findings seem to pave the road to individualized VEGF targeted therapies in mCRC, but more investigations are needed to confirm them. 

### 4.3. Immune Checkpoint Inhibitor Therapy: Monoclonal Antibodies Inhibiting PD-1 

dMMR CRC is hypermutated and expresses numerous neoantigens (frameshift peptides) which induce an active immune microenvironment characterized by abundant tumor infiltrating lymphocytes (TILs) [102]. dMMR tumors are able to evade immune destruction of the vigorous immune system owing to elevated expression of multiple checkpoint proteins including the immune cell co-receptor PD-1, programmed cell death-ligand 1 (PD-L1) and programmed cell death-ligand 2 (PD-L2), cytotoxic T-lymphocyte-associated protein 4 (CTLA-4), lymphocyte-activation gene 3 (LAG-3) and indoleamine (2,3)-dioxygenase (IDO) [103]. Recently, anti-PD-1 (nivolumab and pembrolizumab) antibodies and anti-PD-L1 (MPDL3280A, Medi4736, and BMS-936559) antibodies, namely immune checkpoint inhibitors (ICIs) have been developed to restore T cell activity and are implemented in the treatment of various human malignancies (e.g., melanoma cancer, renal cancer, and non-small cell lung cancer) [104,105,106]. 

There were clinical data demonstrating that dMMR status could predict the clinical benefit of anti-PD-1 therapy in mCRC patients and pMMR mCRC patients might not receive anti-PD-1 therapy in clinic due to the complete absence of objective responses. The anti-PD-1 antibody nivolumab did not demonstrate clinically significant activity in a phase I study of mCRC patients [106]. Only one patient with a dMMR tumor from this cohort, who had a PD-L1 positive tumor displayed complete response after treatment with five doses of nivolumab for six months [107]. A phase II study conducted by Le et al. [108] demonstrated that dMMR CRC patients displayed the immune-related objective response and immune-related progression-free survival (PFS) rates 40% and 78% respectively, compared with pMMR CRC patients 0% and 11%. Thus, the Food and Drug Administration (FDA) approved rapidly pembrolizumab for metastatic/refractory dMMR CRC treatment. dMMR CRC could benefit from immune checkpoint blockade therapy due to high expression levels of checkpoint proteins in the local immune microenvironment [108] and the strong T cell response induced by large amounts of neoantigens [109]. However, only 3%–6% of mCRCs are dMMR phenotype, which indicates that the targeted population in mCRC is very small [110]. Thus the anti-PD-1 therapy might be applied to earlier stage CRC patients to expand its application range [110].

## 5. Prognostic Value of MMR Status in CRC

### 5.1. Prognostic Value of MMR Status in Early Stage CRC

Rich evidence indicated that stage II/III CRC patients with dMMR tumors had a better clinical outcome than those with pMMR tumors [52,111,112]. Bertagnolli et al. [112] reported in their study that CRC patients with dMMR tumors had better 5-year DFS (0.76 vs. 0.67; *p* < 0.001) and OS (0.81 vs. 0.78; *p* = 0.029) than those with pMMR tumors. It was shown that the prognostic effect of dMMR was stronger in stage II than stage III CRC patients [6,7]. In a study by Klingbiel et al. [6] they found that in stage II, relapse-free survival (RFS) and OS were better for CRC patients with pMMR than with dMMR tumors (HR = 0.26; 95% CI = 0.10–0.65; *p* = 0.004 and 0.16; 95% CI = 0.04–0.64; *p* = 0.01). In stage III, RFS was slightly better for dMMR CRC patients (HR = 0.67; 95% CI = 0.46–0.99; *p* = 0.04) [6]. And the better prognosis of dMMR CRC patients might result from a stronger immunologic response driven by abundant TILs in the tumor microenvironment [113]. In addition, several studies confirmed that dMMR CRC had reduced levels of VEGF compared to pMMR CRC [114,115], which might partly explain that patients with dMMR tumors had more favorable prognosis. 

### 5.2. Prognostic Value of MMR Status in mCRC

The very small fraction of dMMR tumors in mCRC patients brought an obstacle to evaluate the prognostic value of dMMR status in mCRC [116]. It was well reported that dMMR was a good prognostic marker in early stage CRC, and several researches showed that dMMR displayed little prognostic value in mCRC. Recently, a nationwide cohort study of 6692 patients suggested that MMR status was not related to survival in mCRC patients [117]. Similarly, Nöpel-Dünnebacke et al. [118] found that dMMR status was not correlated with overall response rate (ORR), PFS and OS in mCRC. Overman et al. [110] found that mCRC patients with dMMR tumors had no improved outcomes and *BRAF* V600E mutation was associated with a poor prognosis in dMMR mCRC. A pooled analysis of four phase III studies demonstrated that compared with those with pMMR tumors, CRC patients with dMMR tumors displayed reduced PFS and OS (HR = 1.33; 95% CI = 1.12–1.57 and HR = 1.35; 95% CI = 1.13–1.61, respectively) which might be driven by *BRAF* V600E mutations [119]. Another study showed that dMMR CRC tumors had significantly poorer survival compared with pMMR CRC tumors (11.1 months vs. 22.1 months, *p* = 0 .017) [94].

## 6. Conclusions and Perspectives

Though the TNM stage remains the key determinant of CRC prognosis and treatment in clinic, there are considerable stage independent inter-individual differences in clinical outcome and therapy response of CRC patients. Hence, prognostic and predictive biomarkers are urgently demanded to accurately inform clinical outcome and guide treatment selection in CRC management. Improved knowledge of the molecular characterization of CRC has allowed the personalized management of this malignancy to advance rapidly. As one of the important molecular characterizations of CRC, dMMR status has been demonstrated as a crucial biomarker for prognosis and response to many drugs used in CRC, which helps the clinicians and patients use medications rationally to avoid dispensable treatment and reduce the burden of patients in CRC therapy. 

The paired MMR deficient/proficient models or the panel of dMMR versus pMMR cell lines were always chosen in preclinical studies. The former models might not take the effects of secondary mutations into account whereas the latter would. In addition, different detection methods for cells apoptosis induced by drugs, such as the clonogenic assay and MTT test were applied in preclinical experiments. All the above factors would lead to inconsistent preclinical results concerning the relationship between the MMR system and the sensitivity to drugs used in CRC treatment. Meanwhile, clinical studies determining the role of MMR status in CRC management were different in scale, whether retrospective or prospective, randomized or not, and different chemotherapy regimens were involved, which might have led to the varied conclusions. Thus, studies in pooled data from similar clinical trials may help to further explore tumor heterogeneity and to highlight the impact of MMR status and other molecular features on prognosis and the efficacy of drugs used to treat CRC. Although dMMR has been shown to provide valuable predictive and prognostic information, it is more important to combine it with other molecular markers such as *KRAS*, *BRAF* V600E mutations [120] or gene expression profiling by next-generation sequencing (NGS) platforms to precisely guide the individualized treatment of CRC [3].

In conclusion, dMMR is a crucial molecular biomarker and plays an important role in CRC management to guide decision-making in clinic. A better understanding of molecular pathways involved in dMMR CRC is pivotal to develop novel therapies and inform suitable therapies of dMMR CRC subsets. With the development of some key scientific discoveries in the molecular biology of CRC, personalized drug therapy of CRC is an undoubted tendency. Although we are a long way from personalized treatment of CRC, we are very optimistic regarding the direction of this field and expect revolutionary progress in the future. 

## Figures and Tables

**Table 1:** Studies evaluating the impact of MMR status on the efficacy of 5-FU-based treatment in CRC.

References	Analyzed/Total	MSI Frequency	Disease Stage	Treatment	Result
Sargent et al. (2010) [53]	457	15%	Stage II & III	FU/LEV or FU/LV vs. No Treatment	Reduced OS in dMMR Tumors Receiving FU-based Adjuvant Therapy (HR, 2.95; 95% CI, 1.02–8.54; *p* = 0.04)
Jover et al. (2009) [60]	505/754	10.1%	Stage II & III	5-FU-based vs. No Treatment	Reduced Survival in dMMR Tumors Receiving FU-based Adjuvant Therapy (pMMR Log Rank *p* = 0.00001; dMMR Log Rank *p* = 0.7)
Tejpar et al. (2009) [61]	1254/3278	22% stage II 12% stage III	Stage II & III	5-FU/FO vs. 5-FU/FO/CPT-11	Prognostic Effect of dMMR in Patients Treated with 5-FU
Sargent et al. (2008) [62]	341	13.8%	Stage II & III	5-FU/LEV, 5-FU/FO vs. No Treatment	Reduced OS (pMMR HR, 0.69; *p* = 0.047; dMMR HR, 1.26; *p* = 0.68) and DFS (pMMR HR, 0.59; *p* = 0.004; dMMR HR, 1.41; *p* = 0.53) in dMMR Tumors Receiving 5-FU-based Adjuvant Therapy
Kim et al. (2007) [56]	542	18.1%	Stage II & III	FU/LV vs. No Treatment	No Difference was Found by dMMR Status.
Westra et al. (2005) [54]	273/391	16%	Stage III	FU-based Chemotherapy	In a Multivariate Model, dMMR Status was not Associated with DFS.
Ribic et al. (2003) [52]	570	16.7%	Stage II & III	5-FU-based Chemotherapy vs. No Treatment	Reduced OS (pMMR HR, 0.72; *p* = 0.04; dMMR HR, 1.07; *p* = 0.80) in dMMR Tumors Receiving 5-FU-based Adjuvant Therapy
Hemminki et al. (2000) [55]	1044	12%	Stage III	5-FU-based Chemotherapy	Improved RFS in dMMR Tumors (*p* = 0.020)
Elsaleh et al. (2000) [57]	656	8.5%	Stage III	5-FU-based Chemotherapy	Better Survival in dMMR Tumors (*p* = 0.0007)

Abbreviations: dMMR, deficient mismatch repair; CRC, colorectal cancer; 5-FU, 5-fluorouracil; FO, folinic acid; CPT-11, irinotecan; LV, leucovorin; LEV, levamisole; PFS, progression-free survival; HR, hazard ratio; MSI, microsatellite instability; OS, overall survival; DFS, disease-free survival; RFS, recurrence-free survival; pMMR, proficient mismatch repair.

**Table 2:** Studies assessing the impact of MMR status on the efficacy of CPT-11 in CRC therapy.

References	Analyzed/Total	MSI Frequency	Disease Stage	Treatment	Result
Tejpar et al. (2015) [6]	1254/3278	21.8% in Stage II 12.1% in Stage III	Stage II/III	5-FU/LV vs. 5-FU/LV/CPT-11	No Difference was Found by dMMR Status
Kim et al. (2011) [81]	197/297	11.7%	mCRC	CPT-11-based Chemotherapy	No Difference in RR and PFS
Bertagnolli et al. (2009) [80]	723/1264	13.3%	Stage III	FU/LV vs. Weekly IFL	Improved Survival in dMMR Patients (*p* = 0.03)
Braun et al. (2008) [82]	931/2135	4.4%	mCRC	Palliative 1st-line 5-FU/CPT-11 or 5-FU/oxaliplatin	No Difference in PFS (HR, 0.93; *p* = 0.7) and OS (HR, 0.66; *p* = 0.2)
Charara et al. (2004) [79]	57	23%	Early Stage Rectal Cancer	5-FU, CPT-11, Radiotherapy and Surgery	Improved Complete RR in dMMR patients
Fallik et al. (2003) [78]	44/72	15.9%	mCRC	CPT-11	Improved RR in dMMR Patients (57% vs. 10.8%; *p* = 0.009)

Abbreviations: mCRC, metastatic colorectal cancer; RR, response rate; IFL, irinotecan, FU and LV.
